# The clinical and cost-effectiveness of the BRinging Information and Guided Help Together (BRIGHT) intervention for the self-management support of people with stage 3 chronic kidney disease in primary care: study protocol for a randomized controlled trial

**DOI:** 10.1186/1745-6215-14-28

**Published:** 2013-01-28

**Authors:** Christian Blickem, Tom Blakeman, Anne Kennedy, Peter Bower, David Reeves, Caroline Gardner, Victoria Lee, Carolyn Chew-Graham, Gerry Richardson, Helen Brooks, Shoba Dawson, Rahena Mossabir, Praksha Jariwala, Angela Swallow, Evan Kontopantelis, Hannah Gaffney, Nicola Small, Eldon Spackman, Anne Rogers

**Affiliations:** 1 Centre for Primary Care, Institute of Population Health, University of Manchester, Oxford Road, M13 9PL, Manchester, UK; 2 Health Sciences, University of Southampton, Highfield Campus, 12 University Road, SO17 1BJ, Southampton, UK; 3 Centre for Health Economics, University of York, YO10 5DD, York, Heslington, UK; 4 Primary Care & Health Services, University of Keele, ST5 5BG, Staffordshire, UK

**Keywords:** Vascular disease, Chronic kidney disease, Self-management, Randomized controlled trial, Social disadvantage, Social networks, Social prescribing, Minimally disruptive medicine

## Abstract

**Background:**

Improving the quality of care for people with vascular disease is a key priority. Chronic kidney disease (CKD) has recently been included as a target condition for general practices to add to registers of chronic conditions as part of the Quality and Outcome Framework. This paper outlines the implementation and evaluation of a self-management intervention involving an information guidebook, tailored access to local resources and telephone support for people with stage 3 chronic kidney disease.

**Methods/Design:**

The study involves a multi-site, longitudinal patient-level randomized controlled trial. The study will evaluate the clinical use and cost-effectiveness of a complex self-management intervention for people with stage 3 chronic kidney disease in terms of self-management capacity, health-related quality of life and blood pressure control compared to care as usual. We describe the methods of the patient-level randomized controlled trial.

**Discussion:**

The management of chronic kidney disease is a developing area of research. The BRinging Information and Guided Help Together (BRIGHT) trial aims to provide evidence that a complementary package of support for people with vascular disease that targets both clinical and social need broadens the opportunities of self-management support by addressing problems related to social disadvantage.

**Trial registration:**

Trial registration reference: ISRCTN45433299

## Background

Vascular disease is the largest single cause of long-term ill health and disability in the United Kingdom and disproportionately affects socially disadvantaged populations [1,2]. Providing effective self-management support is a key policy focus which aims to improve the skills and confidence of patients to manage their illness. However, existing approaches to supporting self-management have shown limitations, particularly with equivocal evidence in outcomes for socially disadvantaged groups [3-5]. The limitations of these approaches to self-management support strategies are that they over-simplify the everyday challenges faced by people living with long-term health problems and have often overlooked the social and structural barriers such as access to resources, identifying needs appropriately, and the role of personal networks of support [3,4,6,7].

The BRinging Information and Guided Help Together (BRIGHT) intervention aims to address this implementation gap and recognizes a necessity for changes to be made at the level of the patient, practice, organization and community [7-12]. With a particular need to address the interface between primary care and resources in the community, the BRIGHT intervention intends to enhance the effectiveness of self-management strategies by focusing more on patient contexts and personal networks that are centrally involved in the mobilization and deployment of resources used in managing chronic disease [13].

The BRIGHT intervention aims to improve the care and outcomes for people with vascular conditions, in particular stage 3 CKD, by:

1. Providing patient information that incorporates both clinical and lay-experiential knowledge.

2. Broadening the scope of self-management support to address both social and clinical needs.

3. Linking patients’ needs and preferences to local community resources.

4. Embedding strategies for self-management support into existing approaches to the delivery of care for patients with long-term conditions.

This approach builds on evidence that socially disadvantaged groups may benefit from interventions which are lay-led and community-located [2,4] and builds on evidence that social networks are implicated in self-care support outside formal health services [6]. A shift in emphasis from an exclusive focus on the individual is required to one which includes a greater focus on the mobilization of resources and interaction with aspects of everyday life (for example, home, family, work, leisure and friends). Therefore the aim of the BRIGHT intervention is to elicit people's needs in order to develop social support strategies that can be added to existing evidence-based approaches to guided self- management support [13].

### Developing a patient information resource

The BRIGHT intervention aims to improve self-management support for people with vascular disease by addressing an identified gap in the provision of information for patients with stage 3 chronic kidney disease (CKD) [14]. A self-management guidebook was developed for this trial entitled ‘Keeping your Kidneys Healthy - a guide to help you understand and manage the early stages of kidney problems’. The development of the guidebook followed principles which have been established as part of a whole systems approach to the provision of self-management support [8,15-18]. These are: lay informed experiences to be given equal weight to medical and clinical informed evidence; the inclusion of personal experiences and anecdotes to bring the information alive and; information which is based on patient’s expressed needs and actual ways of managing and not on assumptions. Further principles followed included: good design; use of plain English; and use of clear diagrams, pictures and cartoons to aid understanding. The content includes and acknowledges areas of uncertainty or lack of evidence to encourage people to think about how they currently manage their condition, what they want to change, and plan how to make changes by themselves using the support of family and friends or working with their doctor or nurse.

Provision of meaningful information around stage 3 CKD may provide a platform for improved medicines management and lifestyle change [19]. CKD is common, is associated with lower socioeconomic status, and often exists with other conditions such as hypertension, diabetes and ischemic heart disease [20-24]. CKD is an independent risk factor for cardiovascular disease and its early recognition and treatment, targeted at reducing blood pressure can prevent or delay progression and reduce the risk of cardiovascular disease and renal failure [25,26]. However, stage 3 CKD is usually asymptomatic and awareness of diagnosis is low [27].

Professionals have expressed concerns that disclosing asymptomatic stage 3 CKD to patients may create anxiety, particularly ‘in the ‘elderly’ and those in whom clinical benefit is deemed less certain [14]. General practitioners (GPs) and practice nurses may not always disclose a diagnosis of CKD to patients as the condition and its associated risks are considered difficult for patients to understand [28]. Furthermore, most qualitative studies on experiences of managing kidney disease have focused on end stage renal failure and dialysis [29,30]. Taking these concerns into account, the BRIGHT intervention aims to improve the delivery of vascular care through the provision of an information resource as a key component of a complex self-management intervention for patients diagnosed with stage 3 CKD. The guidebook was developed to address a tendency to avoid information exchange around CKD and the approach taken aims to avoid treating stage 3 CKD in isolation but rather recognizes its management in the context of everyday life and in which maintaining health while living with multiple conditions is the norm rather than the exception [31].

### Broadening the scope of self-management support: linking patients’ needs and preferences to local community resources

The BRIGHT intervention draws on the notion of ‘social prescribing’ which is a recent initiative embracing an approach to support for people with long-term health problems that links clinical, everyday and personal contexts. Social prescribing is a link scheme between primary care and the community and voluntary sector which provides pathways for patients to access acceptable and available community support [32-35]. There is some evidence that this approach can reduce isolation in patients, increase the confidence of patients to manage their health and improve the patient-clinician consultation by providing alternative community-based options of support for patients, which are acceptable and tailored to specific needs [32,34]. However uncertainties persist about optimal ways to refer patients from primary care into community and voluntary sector organizations because clinicians are either unaware of existing services or are unclear about how they meet the clinical needs of patients.

### Embedding self-management support strategies into current delivery of care

The BRIGHT intervention has been informed by Normalization Process Theory (NPT), which provides a framework to understand and address processes underpinning existing delivery of care [36,37]. The study embraces the concept of *minimally disruptive medicine*, which focuses on improving health outcomes through provision of services that are designed to enhance individual capacity to manage health and reduce the burden of treatment on people’s lives [38]. This approach includes prioritizing care from the patient’s perspective, addressing coordination between services, as well as acknowledging multimorbidity in both the design of care delivery and development of an evidence base. The BRIGHT intervention has therefore been designed to build on dialogue between primary care clinicians and patients.

The BRIGHT trial aims to provide evidence that a complementary package of support for people with vascular disease which targets both clinical and social needs can broaden the scope of self-management support by addressing problems related to social disadvantage [33,34]. In summary, the BRIGHT intervention builds on the evidence in Table 1.

**Table 1:** Components of the BRinging Information and Guided Help Together (BRIGHT) intervention

**Evidence**	**Component of the BRIGHT intervention**
Information can be an effective platform for changing behavior and can be improved by integrating patient experience alongside medical information about management and treatment [4,15-17].	A kidney information guidebook for people with stage 3 CKD.
Social prescribing has shown encouraging results at improving health outcomes, reducing social isolation and improving the quality of the clinical consultation [35].	A self-assessment questionnaire which is linked to an interactive website to tailor access to types of community-based resources.
Socially disadvantaged groups are more likely to benefit from interventions that fit with patients’ existing adaptations and that reduce social isolation and improve access to resources [3-6,39].	A telephone consultation with a support coordinator to guide patients through the questionnaire and website.

Our approach for the design and proposed evaluation of the BRIGHT complex intervention follows guidance as outlined by the Medical Research Council [40]. We are developing an evidence base for the BRIGHT intervention using a mixed methodology combination of a randomized controlled trial (RCT), nested qualitative research and economic evaluation.

### Aims

The principal research question is: In patients with stage 3 chronic kidney disease, can a complex self-management intervention to improve knowledge of CKD management and promote links with local community resources improve self-management capacity, health-related quality of life and blood pressure control compared to care as usual?

## Methods/Designs

### Design overview

The study is a two-arm, patient-randomized, RCT. Participants will be primary care patients registered with general practices with an existing diagnosis of stage 3 CKD. Participants will be allocated to one of two groups: an intervention group or a control group. The intervention group will receive the BRIGHT intervention, to be delivered after a recent clinical appointment with a GP or practice nurse. Participants in the control group will receive care as usual from their general practice and will be provided with the guidebook and website link at the end of the trial. Patient data will be collected at two time points: at baseline (prior to group allocation and intervention delivery) and at six months post-intervention. The reporting of this trial follows the Consolidated Standards of Reporting Trials (CONSORT) guidelines [41-43].

### Population

Patients must have a clinical diagnosis of stage 3 CKD and will be identified from disease registers at GP practices. Patients must be able to communicate in English and there will be agreement with practices that the patient is appropriate to be recruited to the trial. Patients receiving palliative care or who have reduced capacity to consent will be excluded from the trial.

Inclusion criteria will be stage 3 chronic kidney disease (both stages 3a and 3b).

Exclusion criteria will be: mental incapacity to provide informed consent; inability to communicate in English; receiving palliative care. Only one person per household will be eligible for the study to avoid potential contamination between control and intervention patients.

### Intervention

The proposed intervention entails provision of:

**Figure 1:**
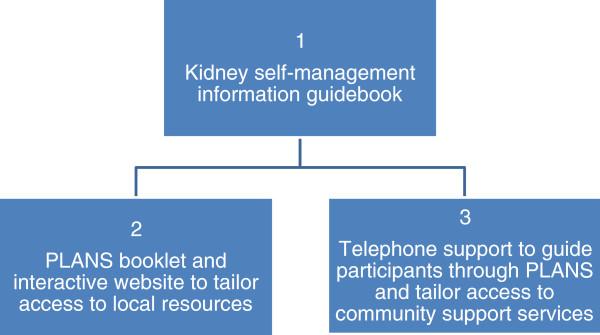
BRinging Information and Guided Help Together (BRIGHT) Intervention.

1. A kidney information guidebook.

2. A PLANS booklet and access to an interactive website with tailored access to local resources.

3. Telephone support from a dedicated peer support worker (see Figure 1).

### The guidebook

The kidney information guidebook builds on previous research conducted by the authors, which shows that health information is an effective strategy to support self-management and can be improved by incorporating the views and experiences of patients with clinical guidance about management and treatment [4,15-17,44].

Two focus groups of patients with stage 3 CKD were conducted and were recorded, transcribed and analyzed. The guidebook was then developed using the empirical data and qualitative literature. It includes sections on patient experience, self-management options and techniques, and the uncertainties and complexities of living with long-term conditions. The guidebook also includes basic information about the early stages of kidney problems and how they are managed by the health service. The development process included input and feedback from clinicians on the clinical guidance included in the guidebook.

### PLANS booklet and website

PLANS (Patient-Led Assessment for Network Support) is an intervention developed from empirical work conducted by this center with patients with vascular disease that aims to increase social contact and promote community support and engagement by creating links with local resources based on need and personal preference. PLANS is a patient self-assessment tool which links patients with vascular disease to relevant local health resources. PLANS aims to support sustainable health choices by promoting local resources that are both acceptable and accessible to patients. The resources in the PLANS database fall into one or more different categories (see Table 2).

**Table 2:** PLANS categories

**Category**	**Description**
*Well-being*	Groups, services and activities intended for general well-being and social participation (for example, hobbies and interest groups).
*Health education*	Groups, services and activities that offer health related advice, guidance and support such as healthy eating or condition-specific support.
*Practical support*	Groups, services and activities that offer everyday practical support (for example, day care and adult respite and home support such as handyman services).
*Diet, cookery and healthy eating*	Groups, services and activities that offer support and guidance for diet and healthy eating (for example, slimming groups).
*Exercise and physical activity*	Groups, services and activities that provide opportunities to keep fit and participate in physical activity (for example, sports clubs or walking groups).
*Mobility*	Services that offer support for people with limited mobility or have difficulty using public transport (for example, shopping and delivery services).

Patients receiving the intervention will have access to the PLANS website and a PLANS booklet will be available for patients without internet access. Both the website and the booklet contain the same information. The telephone support facility will guide participants through the PLANS questions and options on the database.

### Telephone support

Self-management interventions built around structured telephone support offer a mechanism to reach patients who have poor access to health care. Evidence indicates telephone-based support in patients with associated vascular conditions improves patient outcomes, such as social support [45,46], and reduces hospitalizations [47,48]. Non-health care professionals, such as ‘peers’, are increasingly being used to offer telephone support as an alternative form of support from that offered by clinicians to reach disadvantaged populations and to provide more efficient delivery of care [39,49,50]. Peer telephone support may have potential as a cost-effective intervention [51,52]. Evidence suggests that peer telephone support produces better health outcomes including effects on self-management behavior for patients with associated vascular conditions [46,48,49,53].

As part of BRIGHT we will recruit peer volunteers (or lay workers), to deliver structured telephone support to all patients in the intervention group. Peer support workers will participate in a three-hour training session to clarify and review evidence-based content materials required for effective structured telephone support [49,54], and to clarify when and how to facilitate appropriate referrals to local resources. To facilitate training, peer support workers will develop a detailed workbook and receive guidance on how to deliver the intervention by telephone.

Patients in the intervention group will receive two telephone calls from a dedicated peer support worker; one call at one-week post-administration of the kidney information guidebook and the PLANS booklet, followed by another call at four-weeks post-intervention. A peer support worker will guide the patient through options for local organizations and activities using motivational techniques over the telephone. Additionally, patients will be able to discuss alternative options for maintaining general vascular health, lifestyle and self-management support (for example, diabetes-specific education programs) with a peer support worker. Peer telephone support will be made available (Monday to Friday) throughout the course of the trial for ongoing contact by participants, should they require further assistance in linking to community resources.

### Outcomes

All outcomes are at the level of the patient. The primary end-point will be the six-month follow-up for patient health outcomes and costs. The trial has three primary outcomes: one clinical outcome of blood pressure control (dichotomized as controlled versus not controlled) and two patient-reported outcomes of 1) self-management ability, and 2) health-related quality of life (see Additional file 1). Secondary outcomes include additional measures of self-management ability, health status, anxiety (general and CKD-specific), loneliness, medication adherence, social networks and social involvement. Information on service utilization and resource use will also be collected for use in a cost-effectiveness analysis (see below). Each patient will complete a baseline questionnaire, and a follow-up questionnaire will be sent at six months (see Additional file 1).

### Study processes

We aim to recruit patients registered with general practices in Greater Manchester, which participated in a renal collaborative project established by the Collaboration for Leadership in Applied Health Research and Care (CLAHRC) for Greater Manchester [55]. A number of these practices will be in areas of socioeconomic disadvantage as identified by Index of Multiple Deprivation (IMD) scores.

### Patient recruitment and randomisation

Practices will be offered options for how to recruit patients from their CKD disease register to the study. These options will include:

**Figure 2:**
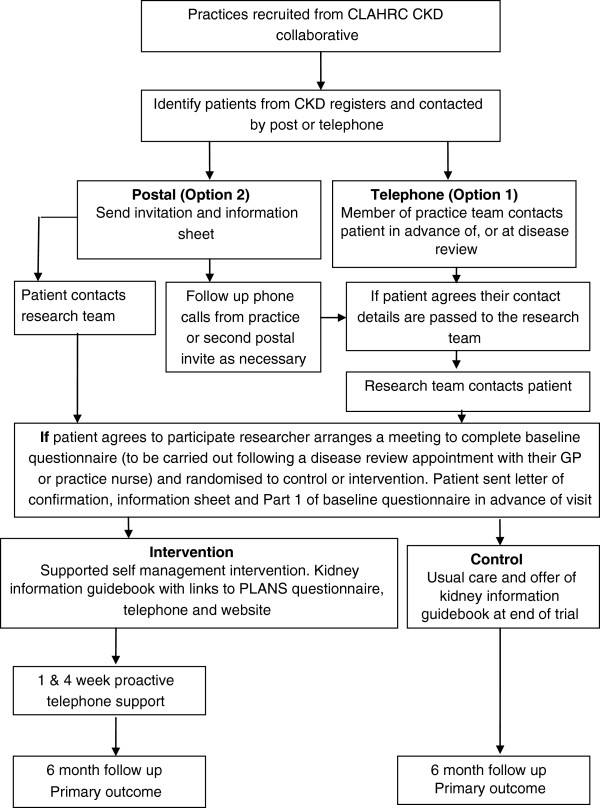
BRinging Information and Guided Help Together (BRIGHT) trial recruitment flow chart.

1. Telephone contact will be made by a member of the practice team to patients due to be seen for a vascular disease review and/or if the GP or nurse prefer, through raising the study at the end of the consultation. These patients will be informed about the BRIGHT trial and asked if their contact details can be forwarded to the BRIGHT team for them to be contacted for further information.

2. Patients due to be seen for a disease review will be sent an invitation and information sheet by their general practice and asked to return a form agreeing to be contacted (see Figure 2).

Patients who agree to participate in the trial will be sent Part 1 of the baseline questionnaire to be completed prior to a meeting with a researcher. A researcher will then arrange to meet with the patient at a time and place convenient for them. Once consented, the researcher will collect Part 1 of the questionnaire and then complete Part 2 of the baseline questionnaire with the patient. The baseline questionnaire is divided in this way to reduce the burden on participants at one time point. In addition, some measures in Part 2 of the baseline questionnaire are optimally delivered at a face-to-face interview because of their relative complexity.

Following completion of the questionnaires, the clinical trials unit will be contacted (by phone or online) and the patient will be allocated to a trial arm via a minimization algorithm (incorporating a random component) to maximize balance between the two arms on key prognostic factors. Minimization will be performed with respect to age, smoking status and evidence of other vascular disease, and will be stratified by practice. Allocation is centralized to prevent the researcher from consciously or unconsciously affecting the patient selection process. This approach will prevent selection bias and will maximize the precision of the treatment effect estimates by ensuring balance between arms with respect to practice recruitment rates and the minimization factors.

This approach will prevent selection bias and will maximize the precision of the estimates of the treatment effects by ensuring balance between arms on the selected key variables. Although observer bias does not apply in this case, researchers are blinded to prevent any chance of them consciously or unconsciously affecting the patient selection process.

The patient will be informed of their allocation to either control or intervention group immediately after the baseline interview. If allocated to the intervention the patient will be given a kidney information guidebook, website information and the PLANS booklet. They will be informed that they will receive a phone call from a peer support worker in one week to discuss the PLANS options in the booklet/ website. Practices will be informed of patient participation in the trial.

### Sample size calculation

The study is designed to have 80% power to detect a standardized effect size of 0.25 on the primary outcome of self-management between the control and intervention arms. This is a relatively small effect, but is in line with most sizes of effect observed in our previous trials of self-management interventions. Using an alpha of 0.05, and assuming a correlation of 0.5 between outcome measures at baseline and follow-up (a conservative estimate), and 25% attrition of participants, we will recruit 500 patients across both arms of the trial. We aim to recruit 16 to 18 practices and to recruit 28 to 32 patients from each practice.

### Analysis

For each of the outcomes, multilevel regression analysis will be used to examine differences between trial arms. For binary outcomes (for example, BP control) the model will be logistic regression; for continuous outcomes the model will be linear regression. All analyses will follow intention-to-treat principles and a pre-specified analysis plan. Primary analyses will control for baseline scores and the design factors (practice, age-group, smoking status, and additional vascular disease). Where appropriate, sensitivity analyses will be conducted (for example, treating all missing BP data as failures; control for additional covariates; bootstrapped *P* values for skewed outcomes). All analysis will be undertaken using Stata version 12 (http://www.stata.com) and an alpha level of 5%. In the case of missing data values, we will apply mean imputation and regression imputation where rates are low, and consider multiple imputation where they exceed 10%.

### Cost-effectiveness analysis

This evaluation will assess the cost-effectiveness of self-management support compared with usual care.

#### Utilization and resource use

Resource use data from primary and secondary care contacts will be collected using patient questionnaire at six month follow-up. Questionnaires previously used in large multicentre RCTs will be adapted to capture resource use.

#### Unit costs

Unit cost estimates from published data sources will be applied to relevant resource use data collected above (for example, cost of GP visit applied to the number of GP visits) to generate a total cost per patient (post-randomization).

#### Intervention costs

We will estimate the cost of developing and producing the booklet and will also assess the time spent training individuals in telephone support.

#### Measurement of effectiveness within the trial

For the trial-based economic evaluation, the measure of effect will be the EQ5D [56]. EQ5D will be collected at baseline and then at six months. This instrument can be used to generate Quality Adjusted Life Years (QALYs). The QALY will be calculated using the Area under the Curve method and adjusting for baseline scores on the EQ5D. In addition, regression analyses will be conducted to identify other factors that may affect individual health state; these may include age, gender and the patient’s primary diagnosis.

#### Extrapolation to the longer term

Changes in Health Related Quality of Life may be identified within the trial period, and if so, these will be identified as part of the within-trial analysis. However, it is also likely that some costs and consequences of the intervention will occur after the completion of trial follow-up. Ideally, the economic evaluation should consider all relevant costs over an appropriate time horizon, and hence we will attempt to build a model to establish the cost-effectiveness of the intervention over the longer time horizon. The generation of a longer term model depends on the existence of links between short-term outcomes, such as BP control, and longer term quality of life. A review of the literature will be conducted to establish whether it is possible to make these links and therefore populate the model. If it is feasible to make links between short- and long-term outcomes, the model will be used to generate costs and Quality Adjusted Life Years (QALYs) over the appropriate time horizon.

#### Synthesis of costs and outcomes

If appropriate, cost and QALY data will be synthesized to generate an Incremental Cost-Effectiveness Ratio (ICER) where additional cost of the intervention is formally compared with additional benefits. The ICER can be used to inform the adoption decision. However, economic evaluation is conducted under conditions of uncertainty, which in this data will be represented using cost-effectiveness acceptability curves (CEACs) generated through the use of probabilistic sensitivity analysis. CEACs will plot the probability of self-management support being cost-effective for a range of threshold values of an additional QALY, and will be presented for both the within-trial analysis and (if feasible), the longer term model.

#### Missing data

The cost-effectiveness analysis will apply imputation techniques to address the statistical issues related to the presence of missing resource use and health outcome data. Imputation of missing data will be conducted using STATA software and will use recommended techniques such as multiple imputation.

#### Sensitivity analysis

Several sensitivity analyses will be performed to determine the robustness of the results to altering certain assumptions. For example, alternative forms of imputation of missing data could alter the assessment of cost-effectiveness.

### Process evaluation

A process evaluation has been designed to complement and provide additional information concerning the trial. Details about the process evaluation and accompanying qualitative study are included in Additional file 2.

### Ethics

The trial has received full ethical approval from the Health Research Authority (REC reference: 11/NW0855) and will be conducted in accordance with the UK Departments of Health’s Research Governance Framework.

Participating practices will be reimbursed for their time and patients will receive a small incentive for participating in the trial. The trial will not disadvantage patients in the intervention or control group in terms of the care received from their GP practice.

## Discussion

The BRIGHT intervention has been designed to improve vascular health through provision of an information resource for patients with stage 3 CKD and to broaden the scope of self-management support by linking with health-relevant community resources. This approach draws on understandings that the hidden ‘work’ associated with long-term condition management is often absorbed by personal networks. Therefore, patients with limited personal resources and who live in socially disadvantaged circumstances disproportionately experience the extra burden of living with chronic illness. The BRIGHT intervention has been designed to utilize personal networks and community contexts as a complementary strategy to support self-management in order to target support more effectively for socially disadvantaged populations.

### Methodological considerations

To date, we are unaware of previous RCTs evaluating interventions aimed at improving the delivery of care for people diagnosed with stage 3 chronic kidney disease. During the design of the trial, there were a number of methodological issues that we needed to consider that are pertinent to the current management of stage 3 CKD in primary care.

### Minimally disruptive medicine

We recognize the potential for information about chronic kidney disease to cause distress in patients. Therefore, the concept of minimally disruptive medicine has provided a framework for the trial design [38], which has sought to reduce the potential for distress. Patients will only be recruited into the trial if they have already been diagnosed by their GP as having stage 3 CKD and are already on a CKD disease register at their general practice. In addition, in order to avoid disruption to routine care, recruitment and six-month follow-up have been aligned with recommended clinical practice for patients with stage 3 CKD. Visits to patients for recruitment will take place within approximately six weeks of their routine 'disease review' appointment with their GP or practice nurse. In doing so, the study aims to build on rather than disrupt existing dialogue between participants and their registered GP or nurse. In addition to initial contact being made by the practice concerning the research, this approach aims to provide further opportunities for the potential participant to raise any queries about the research with their registered health practitioner in advance of a face-to-face meeting with a member of the research team.

### The quality and outcomes framework

Over the past decade there have been changes to the funding structure of UK general practice [57]. In 2004, the Quality and Outcomes framework (QOF) was introduced. Based on best available evidence, indicators of quality have been assigned to certain long-term conditions. Practices are then paid for achieving targets derived from these indicators. Clinical information systems comprised of disease registers and computer templates have been developed to help professionals deliver such care.

CKD has been included within the QOF since 2006. However, although people with stages 1 to 2 with proteinuria are at higher cardiovascular risk than patients with stage 3A and no proteinuria, with the exception of diabetes, QOF only incentivises care for people diagnosed with CKD stages 3 to 5 [20]. We recognize that provision of appropriate information may be relevant for this group of patients but for trial purposes in order to be confident of recruiting patients with a recorded diagnosis, a decision was made to limit recruitment to patients on general practice CKD registers who have a diagnosis of either CKD stage 3A or 3B.

### Registered prevalence of CKD

National QOF data indicates that although incentivised, CKD stages 3 to 5 are frequently unrecognized [58]; hence we aim to recruit patients registered with general practices in Greater Manchester that participated in a renal collaborative project established by the Collaboration for Leadership in Applied Health Research and Care (CLAHRC) for Greater Manchester [55]. The aim of the collaborative was to improve the identification of CKD cases in primary care, to reduce the gap between the achieved and expected prevalence of CKD on practice registers by 50%, and for 75% of those registered CKD patients to achieve NICE Blood Pressure (BP) targets by July 2010. Therefore prevalence of CKD is likely to be higher in ‘collaborative’ practices than in other practices in Greater Manchester and closer to the expected prevalence. Recruiting these practices within the trial will increase the chances of recruiting sufficient numbers of patients into the trial. Also, there is variation in the levels of blood pressure control in these practices (between 51% and 91% of patients achieving target BPs) [55]. However, we recognize that in recruiting participants from these particular practices, the levels of blood pressure control may be higher in the study sample than in the general population.

In summary, self-management support is widely available and evidenced for vascular conditions such as diabetes and heart disease but much less attention has been given to the importance of chronic kidney disease in the context of maintaining vascular health. The BRIGHT intervention has been developed with the understanding that self-management support is a key strategy for supporting people with vascular disease but has a tendency to overlook the importance of everyday life contexts and how these influence the ability of people to manage their health.

## Trial status

At time of submission the BRIGHT trial is in the process of recruiting patients to the study.

## Abbreviations

BP: Blood pressure;BRIGHT: BRinging Information and Guided Help Together;CEACs: Cost-effectiveness acceptability curves;CKD: Chronic kidney disease;CLAHRC: Collaboration for Leadership in Applied Health Research and Care;CONSORT: Consolidated Standards of Reporting Trials;GPs: General practitioners;ICER: Incremental cost-effectiveness ratio;IMD: Index of Multiple Deprivation;NPT: Normalization process theory;QALYs: Quality adjusted life years;QOF: Quality and outcomes framework;RCT: Randomized controlled trial

## Competing interests

The authors declare that they have no competing interests.

## Authors’ contributions

CB and TB wrote the first draft of the paper and all other authors read and approved the final manuscript. DR, EK and PB developed the trial analysis and GR and ES developed the economic analysis. CB, AK, TB, PJ, AR and CCG developed the BRIGHT trial interventions. AR, CCG, AK, TB and CB developed the process evaluation.

## Supplementary Material

Additional file 1 **Outcome measures.** Details of the outcome measures used in the trial [13,59-77]. Click here for file

Additional file 2 **Process evaluation.** Outline of the process evaluation [78]. Click here for file

## References

[B1] Department of Health Vascular Disease Briefing Pack 2009 http://www.dh.gov.uk/en/Publicationsandstatistics/Publications/PublicationsPolicyAndGuidance/DH_098627

[B2] Glazier R Bajcar J Kennie NR Willson K A systematic review of interventions to improve diabetes care in socially disadvantaged populations Diabetes Care 2006 29 1675 1688 10.2337/dc05-1942 16801602

[B3] Rogers A Kennedy A Bower P Gardner C Gately C Lee V Reeves D Richardson G The United Kingdom Expert Patients Programme: results and implications from a national evaluation Med J Australia 2008 189 S21 S24 19143586 10.5694/j.1326-5377.2008.tb02205.x

[B4] Blickem C Bower P Protheroe J Kennedy A Vassilev I Sanders C Kirk S Chew-Graham C Rogers A The role of information in supporting self-care in vascular conditions: a conceptual and empirical review Health Soc Care Community 2011 19 449 459 10.1111/j.1365-2524.2010.00975.x 21158998

[B5] Dutta-Bergman M Developing a profile of consumer intention to seek out additional information beyond a doctor: the role of communicative and motivation variables Health Commun 2005 17 1 16 10.1207/s15327027hc1701_1 15590339

[B6] Vassilev I Rogers A Sanders C Kennedy A Blickem C Protheroe J Bower P Kirk S Chew-Graham C Morris R Social networks, social capital and chronic illness self-management: a realist review Chronic Illness 2011 7 60 86 10.1177/1742395310383338 20921033

[B7] Kennedy A Rogers A The needs of others: self-management skills training and the differing priorities of gay men and asylum seekers with HIV Heal Sociol Rev 2009 18 145 158 10.5172/hesr.18.2.145

[B8] Kennedy AP Rogers AE Bower P Support for self care for patients with chronic disease Br Med J 2007 335 968 970 10.1136/bmj.39372.540903.94 17991978 PMC2071971

[B9] Wagner EH Austin BT Davis C Hindmarsh M Schaefer J Bonomi A Improving chronic illness care: translating evidence into action Health Aff 2001 20 64 78 10.1377/hlthaff.20.6.64 11816692

[B10] Kennedy AP Robinson A Hann M A cluster-randomised controlled trial of a patient-centred guidebook for patients with ulcerative colitis: effect on knowledge, anxiety and quality of life Health Soc Care Commun 2003 11 64 72 10.1046/j.1365-2524.2003.00399.x 14629234

[B11] Kennedy AP Nelson E Reeves D Richardson G Roberts C Robinson A A randomized controlled trial to assess effectiveness and cost of a patient orientated self-management approach to chronic inflammatory bowel disease Gut 2004 53 1639 1645 10.1136/gut.2003.034256 15479685 PMC1774266

[B12] Robinson A Lee V Kennedy A Middleton L Rogers A Thompson DG A randomised controlled trial of self-help interventions in patients with a primary care diagnosis of irritable bowel syndrome Gut 2006 55 5 643 648 10.1136/gut.2004.062901 16099784 PMC1856107

[B13] Rogers A Vassilev I Sanders C Kirk S Chew-Graham C Kennedy A Protheroe J Bower P Blickem C Reeves D Kapadia D Brooks H Fullwood C Richardson G Social networks, work and network-based resources for the management of long-term conditions: a framework and study protocol for developing self-management support Implement Sci 2011 6 56 10.1186/1748-5908-6-56 21619695 PMC3120720

[B14] Blakeman T Protheroe J Chew-Graham C Rogers A Kennedy A Understanding the management of early-stage chronic kidney disease in primary care: a qualitative study Br J Gen Pract 2012 62 e233 e242 10.3399/bjgp12X636056 22520910 PMC3310029

[B15] Kennedy A Robinson A Rogers A Incorporating patients’ views and experiences of life with IBS in the development of an evidence based self-help guidebook Patient Educ Couns 2003 50 303 310 10.1016/S0738-3991(03)00054-5 12900104

[B16] Kennedy A Rogers A Improving patient involvement in chronic disease management: the views of patients, GPs and specialists on a guidebook for ulcerative colitis Patient Educ Couns 2002 47 257 263 10.1016/S0738-3991(01)00228-2 12088604

[B17] Kennedy AP Robinson JA Thompson DG Development of a guidebook to promote patient participation in the management of ulcerative colitis Health Soc Care Commun 1999 7 177 186 10.1046/j.1365-2524.1999.00174.x 11560632

[B18] Protheroe J Rogers A Kennedy AP Macdonald W Lee V Promoting patient engagement with self-management support information: a qualitative meta-synthesis of processes influencing uptake Implement Sci 2008 3 44 10.1186/1748-5908-3-44 18851743 PMC2575203

[B19] Abdi Z Gallagher H Donoghue D Telling the truth: why disclosure matters in chronic kidney disease Br J Gen Pract 2012 62 172 173 10.3399/bjgp12X635958 22520891 PMC3310009

[B20] De Lusignan S Gallagher H Stevens P Harris K Dmitrieva O Tahir A Rafi I Tomson C O' Donoghue D Chronic kidney disease frequently asked questions 2011 London: NHS Employers and BMA http://www.nhsemployers.org/SiteCollectionDocuments/Chronic_kidney_disease_FAQs%20-%20ja040711.pdf

[B21] Stevens PE O'Donoghue D de Luignan S Van Vlymen J Klebe B Middleton R Hague N New J Farmer CK Chronic Kidney Disease management in the United Kingdom: NEOERICA project results Kidney Int 2007 72 92 99 10.1038/sj.ki.5002273 17440495

[B22] Chadban SJ Briganti EM Kerr PG Dunstan DW Welborn TA Zimmet PZ Atkins RC Prevalence of kidney damage in Australian adults: the AusDiab kidney study J Am Soc Nephrol 2003 14 S131 S138 10.1097/01.ASN.0000070152.11927.4A 12819318

[B23] Coresh J Selvin E Stevens LA Prevalence of chronic kidney disease in the United States JAMA 2007 298 17 2038 2047 10.1001/jama.298.17.2038 17986697

[B24] Hossain MP Palmer D Goyder E El Nahas AM Social deprivation and prevalence of chronic kidney disease in the UK: workload implications for primary care Int J Med 2012 105 167 175 10.1093/qjmed/hcr153 21964722

[B25] Matsushita K van der Velde M Astor BC Woodward M Levey AS de Jong PE Coresh J Gansevoort RT Chronic Kidney Disease Prognosis Consortium Association of estimated glomerular filtration rate and albuminuria with all-cause and cardiovascular mortality in general population cohorts: a collaborative meta-analysis Lancet 2010 375 2073 2081 20483451 10.1016/S0140-6736(10)60674-5 PMC3993088

[B26] National Institute for Clinical Excellence Chronic kidney disease early identification and management of chronic kidney disease in adults in primary and secondary care 2008 London: National Institute for Health and Clinical Excellence 25340245

[B27] McIntyre MJ Fluck R McIntyre C Taal M Treatment needs and diagnosis awareness in primary care patients with chronic kidney disease Br J Gen Pract 2012 62 e227 e232 22520909 10.3399/bjgp12X636047 PMC3310028

[B28] Crinson I Gallagher H Thomas N de Lusignan S How ready is general practice to improve quality in chronic kidney disease? A diagnostic analysis Br J Gen Pract 2010 60 403 409 10.3399/bjgp10X502100 20529495 PMC2880740

[B29] Molzahn A Bruce A Sheilds L Learning from stories of people with chronic kidney disease Nephrol Nurs J 2008 35 1 13 20 18372759

[B30] Tong A Sainsbury P Chadban S Walker RG Harris DC Carter SM Hall B Hawley C Craig JC Patients' experiences and perspectives of living with CKD Am J Kidney Dis 2009 53 689 700 10.1053/j.ajkd.2008.10.050 19216015

[B31] Salisbury C Multimorbidity: redesigning health care for people who use it Lancet 2012 380 9836 7 9 10.1016/S0140-6736(12)60482-6 22579042

[B32] Year of Care Thanks for the Petunias – a guide to developing and commissioning non-traditional providers to support the self management of people with long termz conditions 2011

[B33] South J Higgins TJ Woodall J White SM Can social prescribing provide the missing link? Prim Health Care Res Develop 2008 9 4 310 318 10.1017/S146342360800087X

[B34] Brandling J House W Social prescribing in general practice: adding meaning to medicine Br J Gen Pract 2009 59 563 454 456 10.3399/bjgp09X421085 19520038 PMC2688060

[B35] Woodall J South J The Evaluation of the CHAT Social Prescribing Scheme in Bradford South & West PCT 2005 Leeds, England: Centre for Health Promotion Research: Leeds Metropolitan University

[B36] May C Finch T Implementing, embedding and integrating practices: an outline of normalization process theory Sociology 2009 43 535 554

[B37] May C Ballini L Finch T Macfarlane A Murray E Mair F Rapley T Treweek S Normalization Process Theory On-line Users’ Manual and Toolkit 2010 [accessed 5th September 2011]; Available from: http://normalizationprocess.org

[B38] May C Montori V Mair F We need minimally disruptive medicine Br Med J 2009 339 485 487 10.1136/bmj.b2803 19671932

[B39] Batik O Phelan EA Walwick JA Wang G LoGerfo JP Translating a community-based motivational support program to increase physical activity among older adults with diabetes at community clinics: a pilot study of Physical Activity for a Lifetime of Success (PALS) Prev Chronic Dis 2008 5 1 1 7 PMC2248781 18082007

[B40] Medical Research Council Developing and evaluating complex interventions: new guidance MRC 2008 London

[B41] Begg C Cho M Eastwood S Horton R Moher D Olkin I Pitkin R Rennie D Schulz KF Simel D Stroup DF Improving the quality of reporting of randomized controlled trials. The CONSORT statement J Am Med Assoc 1996 276 637 639 10.1001/jama.1996.03540080059030 8773637

[B42] Moher D Schulz KF Altman DG for the CONSORT Group The CONSORT statement: revised recommendations for improving the quality of reports of parallel group randomized trials Ann Intern Med 2001 134 657 662 11304106 10.7326/0003-4819-134-8-200104170-00011

[B43] Schultz KF Altman DG Moher D CONSORT Group Updated guidelines for reporting parallel group randomised trials Int J Surg 2011 9 672 677 10.1016/j.ijsu.2011.09.004 22019563

[B44] Bower P Kennedy A Reeves D Rogers A Blakeman T Chew-Graham C Bowen R Eden M Gardner C Hann M Lee V Morris R Protheroe J Richardson G Sanders C Swallow A Thompson D A cluster randomised controlled trial of the clinical and cost-effectiveness of a 'whole systems' model of self-management support for the management of long- term conditions in primary care: trial protocol Implement Sci 2012 7 7 10.1186/1748-5908-7-7 22280501 PMC3274470

[B45] Heisler M Different models to mobilise peer support to improve diabetes self-management and clinical outcomes: evidence, logistics, evaluation considerations and needs for future research Fam Pract 2009 27 i23 i32 19293400 10.1093/fampra/cmp003 PMC2902359

[B46] Dale J Telephone peer-delivered intervention for diabetes motivation and support: the telecare exploratory RCT Patient Educ Couns 2009 75 1 91 98 10.1016/j.pec.2008.09.014 19013741

[B47] Chen SH Tsai YF Sun CY Wu IW Lee CC Wu MS The impact of self-management support on the progression of chronic kidney disease – a prospective randomized controlled trial Nephrol Dial Transplant 2011 26 3560 3566 10.1093/ndt/gfr047 21414969

[B48] Carroll DL Rankin SH Cooper BA The effects of a collaborative peer advisor/advanced practice nurse intervention: cardiac rehabilitation participation and rehospitalization in older adults after a cardiac event J Cardiovasc Nurs 2007 22 4 313 319 17589284 10.1097/01.JCN.0000278955.44759.73

[B49] Parry MJ Watt-Watson J Hodnett E Tranmer J Dennis CL Brooks D Cardiac Home Education and Support Trial (CHEST): a pilot study Can J Cardiol 2009 25 12 e393 e398 10.1016/S0828-282X(09)70531-8 19960132 PMC2807834

[B50] Kim S Koniak-Griffin D Flaskerund JH Guarnero PA The impact of lay health advisors on cardiovascular health promotion J Cardiovasc Nurs 2004 19 192 199 15191262 10.1097/00005082-200405000-00008

[B51] Wu CJJ Chang AM Courtney M Kostner K Peer supporters for cardiac patients with diabetes: a randomised controlled trial Int Nurs Rev 2012 59 3 345 352 10.1111/j.1466-7657.2012.00998.x 22897185

[B52] Dale J Caramlau IO Lindenmeyer A Williams SM Peer support telephone calls for improving health (Review) Cochrane Database Syst Rev 2008 4 Oct 8 10.1002/14651858.CD006903.pub2 PMC7386897 18843736

[B53] Samuel-Hodge CD Keyserling TC Park S Johnston LF Gizlice Z Bangdiwala SI A randomized trial of a church-based diabetes self-management program for African Americans with type 2 diabetes Diabetes Educ 2009 35 3 439 454 10.1177/0145721709333270 19383882

[B54] Heisler M Vijan S Makki F Piette JD Diabetes control with reciprocal peer support versus nurse care management Ann Intern Med 2010 153 507 515 20956707 10.7326/0003-4819-153-8-201010190-00007 PMC4117390

[B55] National Institute for Health Research Collaboration for Leadership in Applied Health Research and Care (CLAHRC) for Greater Manchester: phase1 The CLAHRC Chronic Kidney Disease Collaborative: improving care for people with chronic kidney disease: report on phase 1 of the CKD Collaborative September 2009–September 2010) 2010 Manchester: CLAHRC http://clahrcgm.nihr.ac.uk/projects/implementation/chronic-kidneydisease# (accessed 16 february 2012

[B56] Euroqol Copyright Group: Euroqol - a new facility for the measurement of health related quality of life Health Policy 1990 16 199 208 10109801 10.1016/0168-8510(90)90421-9

[B57] Department of Health Investing in general practice: the new General Medical Services Contract 2003 London: Department of Health

[B58] NHS Information centre Quality and outcomes framework achievement data 2010/11 and http://www.ic.nhs.uk/webfiles/publications/002_Audits/QOF_2010-11/QOF_Achievement_and_Prevalence_Bulletin 2010_11_v1.0.pdf (accessed 9 September 2012)

[B59] Osborne RH Elsworth GR Whitfield K The Health Education Impact Questionnaire (heiQ): an outcomes and evaluation measure for patient education and self-management interventions for people with chronic conditions Patient Educ Couns 2007 66 2 192 201 10.1016/j.pec.2006.12.002 17320338

[B60] Kind P The EuroQoL instrument: an index of health-related quality of life, in Quality of Life and Pharmacoeconomics in Clinical Trials 1996 Philadelphia: LippincottRaven

[B61] Toobert D Hanson S Glasgow R The summary of diabetes selfcare activities measure Diabetes Care 2000 23 7 943 950 10.2337/diacare.23.7.943 10895844

[B62] Zigmond AS Snaith RP The hospital anxiety and depression scale Acta Psychiatr Scand 1983 67 361 370 10.1111/j.1600-0447.1983.tb09716.x 6880820

[B63] Broadbent E Petrie KJ Main J Weinman J The brief illness perception questionnaire J Psychosom Res 2006 60 631 637 10.1016/j.jpsychores.2005.10.020 16731240

[B64] Ware JE Sherbourne CD The MOS 36 item Short Form health survey (SF36): I. Conceptual framework and item selection Medical Care 1992 30 6 473 483 10.1097/00005650-199206000-00002 1593914

[B65] Russell DW UCLA loneliness scale (Version 3): reliability, validity, and factor structure J Pers Assess 1996 66 1 20 40 10.1207/s15327752jpa6601_2 8576833

[B66] Morisky DE Green LW Levine DM Concurrent and predictive validity of a self-reported measure of medication adherence Medical Care 1986 24 1 67 74 10.1097/00005650-198601000-00007 3945130

[B67] Pescosolido BA Levy J Cabassa LJ, Pescosolido BA The role of social networks in health, illness, disease and healing: the accepting present, the forgotten past, and the dangerous potential for a complacent future Social Networks and Health 2002 New York: JAI

[B68] Petrou S Kupek E Social capital and its relationship with measures of health status: evidence from the health survey for England 2003 Health Econ 2008 17 127 143 10.1002/hec.1242 17516583

[B69] Bajekal M Purdon S Social capital and social exclusion: development of a condensed module for the Health Survey for England 2011 London http://www.dh.gov.uk/prod_consum_dh/groups/dh_digitalassets/@dh/@en/documents/digitalasset/dh_4058955.pdf

[B70] Fiori KL Antonucci TC Cortina KS Social network typologies and mental health among older adults Journal of Gerontology Series B, Psychological Sciences and Social Sciences 2006 61 1 P25 P32 10.1093/geronb/61.1.P25 16399938

[B71] Strauss A Fagerhaugh S Suczek B Weiner C Sentimental work in the technologized hospital Sociology of Health and Illness 1982 4 3 254 278 10.1111/1467-9566.ep10487954 10260459

[B72] Strauss A Work and the division of labor Sociol Q 1985 26 1 19 10.1111/j.1533-8525.1985.tb00212.x

[B73] Corbin J Strauss A Managing chronic illness at home: three lines of work Qual Sociol 1985 8 224 247 10.1007/BF00989485

[B74] Kennedy AP Reeves D Bower P Lee V Middleton E Richardson G Gardner C Gately C Rogers A The effectiveness and cost effectiveness of a national lay-led self care support programme for patients with long-term conditions: a pragmatic randomised controlled trial J Epidemiol Community Health 2007 61 3 254 261 10.1136/jech.2006.053538 17325405 PMC2652924

[B75] Lorig K Stewart A Ritter P Gonzalez V Laurent D Lynch J Outcome measures for health education and other health care interventions 1996 Thousand Oaks: Sage

[B76] Van Der Gaag M Snijders TAB The Resource Generator: social capital quantification with concrete items Social Networks 2005 27 1 29 10.1016/j.socnet.2004.10.001

[B77] Adler NE Epel ES Castellazzo G Ickovics JR Relationship of subjective and objective social status with psychological and physiological functioning: preliminary data in healthy white women Health Psychol 2000 19 6 586 592 11129362 10.1037//0278-6133.19.6.586

[B78] Pahl R Spencer L Phillipson GAC, Morgan D Capturing personal communities, Social networks and social exclusion 2004 Aldershot, UK: Ashgate

