# Supplementary material for: The clinical and cost-effectiveness of the BRinging Information and Guided Help Together (BRIGHT) intervention for the self-management support of people with stage 3 chronic kidney disease in primary care: study protocol for a randomized controlled trial
Source: Trials. 2013 Jan 28;14:28. doi: 10.1186/1745-6215-14-28 (PMC3599273; doi:10.1186/1745-6215-14-28)
Supplement: Additional file 1 — Outcome measures. Details of the outcome measures used in the trial [13,59-77]. [file 1745-6215-14-28-S1.docx]

**Additional file 1**

**Primary outcomes**

**Self management:** ‘The Positive and Active Engagement in Life’ domain from the health education impact questionnaire (heiQ) will be used to measure self-management [[58](#_ENREF_58)].

This will assess how participants’ motivation to engage in life may result in positive outcomes entailing a change of lifestyle and life related activities.

**Health related quality of life (baseline and 6mfu)**

The EQ5D is a generic health related quality of life measure that allows the generation of Quality Adjusted Life Years. The 5 item scale covers mobility, self care, usual activities, pain, anxiety and depression, each with three levels of severity. The EQ5D provides measure of health status based on population tariff where zero equates to dead and one equates to full health [[59](#_ENREF_59)]. QALYs are a measure of health generated from the product of health status and time spent in that health state.

**Blood pressure control:** Collected from practice record, readings taken at prior to baseline and 6 month review appointments, questionnaire data to be collected within 6-8 weeks of the review appointments.

**Secondary outcomes**

**Self-management:** Five of the six domains of the heiQ (Health education and impact questionnaire) [[58](#_ENREF_58)] will be used to measure participant’s ability to self-manage, namely: *social integration and support*; *skill and technique acquisition; emotional wellbeing; self monitoring and insight; and health service navigation*. These domains will assess how; patients socially engage and acquire other support through interaction and its positive impact on them; their use of skills and techniques to manage symptoms and health problems; their overall negative responses including anxiety, depression and anger attributed to their health condition; participants monitor their condition and how their responses (both emotional and physical) lead to actions to self-manage; and how they communicate and negotiate with a range of healthcare professionals and health organisation to get their needs met.

Participant’s health directed behaviour will be measured using the Summary of Diabetes Self-Care Activities (SDSCA). This scale will assess areas of general and specific diet, exercise and smoking behaviour in the last seven days [[60](#_ENREF_60)].

**General anxiety**

The anxiety subscale from the Hospital Anxiety and Depression scale (HADS) will be used to assess whether the BRIGHT intervention alters the general level of anxiety in patients [[61](#_ENREF_61)].

**CKD specific anxiety (6mfu only)**

A disease specific adaptation of an item from The Brief Illness Perceptions Questionnaire [[62](#_ENREF_62)] in conjunction with the HADS will be used to detect CKD-specific anxiety.

**Health status:**

We include a selection of sub-scales relating to physical and mental health status from the Medical Outcomes Survey that have performed well in our previous self-management trials: general health; social/role limitations; energy and vitality; and well-being [[63](#_ENREF_63)].

**Loneliness:**

The UCLA Loneliness Scale (9 items) will be used to assess changes in social contact resulting from social interventions (PLANS) [[64](#_ENREF_64)].

**Medication Adherence**

We are using an adapted version of the Self-reported Measure of Medication-taking Scale [[65](#_ENREF_65)], to check for medication adherence between the two arms of the study at the 6 month follow up stage.

**Social Capital**

Research indicates that social capital may have an influence on care pathways and management of illness [[66](#_ENREF_66)]. All three domains of social capital (social networks, neighbourhood attachment & civic participation) are significantly associated with poor measures of health status [[67](#_ENREF_67)]. Social capital will be measured with [11] items from the Health Survey for England (2003) [[68](#_ENREF_68)].

**Social Networks: (baseline and follow up measure)**

Participants record the members in their social network using a grid adapted from the concentric circles diagram developed by Fiori and colleagues [[69](#_ENREF_69)]. In response to the question, “Who do you think is most important to you in relation to managing your condition?” network members are listed in order of perceived importance. Participants can list as many network members as they wish, including any kind of ‘member’ (eg social group, organisation, pet) they consider important. Interviewees are then asked about their relationship to each member, including number of years known, distance away, frequency of contact. Interviewees then rate the amount of ‘work’ each network member contributes on a five-point scale from ‘None’ to ‘A lot’ in the following three domains: *Illness work, practical everyday work and emotional work* [[13](#_ENREF_13), 70,71,72].

**Service utilisation:**

We will utilise a modification of the service utilisation questionnaire from the national evaluation of the Expert Patients Programme which measures primary health care (GP visits, GP practice visits, pharmacy), community health and social care, secondary health care services, out of pocket costs and costs of lost productivity [[73](#_ENREF_73)]. Unit cost estimates will then be applied to these resource use data to generate patient level cost estimates. We will also estimate the cost of developing and delivering the intervention

**Glycaemic control (HbA1c)**: (for patients with diabetes only)

HbA1c measures at baseline and 6 month follow up will be obtained from the practice electronic records using the same procedure as for blood pressure control (see above).

**Additional measures**

Some additional measures will be included in the questionnaires (baseline and six months follow up) as possible moderators of effect.

**General Health (baseline and 6mfu)**

A single item scale from the Stanford Chronic Disease Self-Management study will be used to measure participant’s general health. Participants will be asked to rate their general health with options ranging from ‘excellent’ to ‘poor’ [[74](#_ENREF_74)].

**Normalisation (baseline and 6mfu)**

Informed by the Normalisation Process Theory a four-item scale was developed by this research team to measure how participants were managing their long term condition and the extent to which it affects them and other people around them.

**Employment situation**

Both at baseline and six months follow up participants will be asked to state their ‘current work’ situation. We are asking this question at six months to determine if the PLANS intervention has any positive impact on them (i.e. uptake of any voluntary position).

**Satisfaction with health-related community groups & services (baseline and 6mfu)**

Participants will be asked to list the names and the frequency of use (in the last 6 months) of any community groups, services or activities that they feel may be important to their health and well-being. This item will be used both at baseline and at six months follow up as it will facilitate in identifying any changes resulting from the uptake of social intervention (PLANS). We also asked participants to rate their levels of satisfaction with opportunities to take part in various health related groups and services.

A single item will be used to assess if participants are involved in activities related to various organisations including Political groups, community action groups, community activity or social club or other groups or organisations. Additionally, participants will be asked to rate their levels of satisfaction with opportunities to participate in these groups/organisations [[67](#_ENREF_67)].

**Resource generator (baseline and 6 mfu)**

The resource generator will be utilised as a further measure of social capital which conceptualises social networks and relationships as personal resources [[75](#_ENREF_75)].

**Perceptions of local neighbourhood (baseline and 6 mfu)**

Research evidence suggests that social capital and its domains (social network, attachment to neighbourhood and civic participation) are notably related to poor measures of health status. The impact of social capital on participant’s health status will be measured using items from the Health Survey of England [[67](#_ENREF_67)].

**Trust and helpfulness (baseline and 6mfu)**

Participant’s trust in other people was measured using two variables ‘helpful’ and ‘advantage’ of the ‘trust domain’ [[67](#_ENREF_67)].

**Health related internet use (baseline and 6mfu)**

A single item will be used to identify if participants used the internet to look for health related medical information (i.e. causes, symptoms, treatment) or support.

**Subjective social status (baseline)**

A 10 rung ladder diagram will be utilised to measure participant’s subjective social status [[76](#_ENREF_76)].
