# Supplementary material for: The clinical and cost-effectiveness of the BRinging Information and Guided Help Together (BRIGHT) intervention for the self-management support of people with stage 3 chronic kidney disease in primary care: study protocol for a randomized controlled trial
Source: Trials. 2013 Jan 28;14:28. doi: 10.1186/1745-6215-14-28 (PMC3599273; doi:10.1186/1745-6215-14-28)
Supplement: Additional file 2 — Process evaluation. Outline of the process evaluation [78]. [file 1745-6215-14-28-S2.docx]

**Additional file 2**

**BRIGHT Process evaluation**

As part of the BRIGHT trial a nested longitudinal qualitative study will be conducted to explore the influences on processes and outcomes of the BRIGHT intervention and to understand factors that may bring about change to the individual and community management of stage 3 chronic kidney disease. The primary focus is on engagement with and implementation of key elements of the BRIGHT intervention as important outcomes to assess. We will explore the way in which the intervention changes existing adaptations and interactions with others or the extent to which these stay the same. The qualitative research aims to explain trial results with reference to the use of resources and personal networks within which people with stage 3 CKD live their everyday lives. A further focus is on networks of support and the extent to which these change or stay the same as a result of the introduction of a self management intervention. The BRIGHT trial raises specific points of learning for patients concerning 1) context (primary care/community), 2) the early stage of the condition, 3) the co-existence of other conditions (i.e. multimorbidity) and 4) the connections people make as a dimension of self-management support.

**Methods**

Semi-structured interviews and personal network ‘concentric circles’ methods [[77](#_ENREF_77)] will focus on exploring the role of individual network members in chronic illness management with a focus on participants’ management of long-term conditions and how resource access, social networks and relationships are implicated for CKD and associated vascular condition management.

**Sampling**

Interviews will be undertaken with between 15-20 individuals in the experimental group of the BRIGHT. Purposeful sampling of those who have indicated on the consent form that they are willing to be contacted for a face to face interview will include: a broad range of respondents from a range of practice locations. Sampling in Rounds 2 and 3 of interviewing (see below) will be based on types of resources accessed as well as based on outcomes (both positive and negative).

**Data Collection**

Interviews will be conducted at three time points during the course of the BRIGHT trial. At all stages, the interviews will be audio-taped with participants’ consent and transcribed verbatim.

***Round 1***

15 interviews to be conducted prior to or at the time of recruitment. Topics to be explored during these initial interviews will include:

1. Burdens of living with illness and of management
2. Existing self-management strategies
3. Chronic illness work, networks and connections to resources. Awareness, knowledge of existence and implications of relationship between a) existing non CKD conditions and CKD, b) services in primary and secondary care and c) CKD and self-management.
4. Lay patient conceptualisation of access, engagement and use of primary care services for CKD and related conditions including CDM.
5. Lay knowledge about the cause of the conditions, discovering disclosure of conditions (in general) and experience of disclosure for existing condition and CKD specifically

***Round 2***

Telephone interviews to be conducted with the 15 participants from Round 1, plus an additional purposeful sample of 10 people who have consented to further contact and who have experience of use the Patient Led Assessment tool for Network Support (PLANS). These interviews will be conducted 6 months post-introduction to the intervention. Topics to be explored will include:

1. Experience of and response to use of kidney self-management guidebook, guided self-management, menu plans, use of engagement with primary and secondary care as well as connections in the community.
2. Mapping changes to personal self-management strategies for vascular disease.
3. Disclosure of CKD – added burden or helpful process, have attitudes to disclosure changed
4. Concentric circle check – has anything changed from the original mapping

***Round 3***

Using quantitative data from the BRIGHT trial, we will sample those with positive outcomes as well as those where outcomes are neutral or negative. Identifying what works for whom and in what context and why is the focus of Round 3 of interviewing. Topics to be explored include:

1. The mobilisation and use of resources through use of the 12 month follow up with concentric circles.
2. In-depth contact and engagement with health professionals.

**Qualitative data analysis**

The first stage of analysis will identify responses to original themes (Round 1) and identify emergent themes for follow up in Round 2. This analysis will be informed by adapted framework analysis. In the final round of analysis, interviews from all stages will be read ‘back to back’ for individual respondents. We will use netdraw to map the network and illness work properties of individuals. We will compare consistency and differences within respondent’s accounts as well as between respondent’s accounts using constant comparative method to identify and unite overarching themes in the data. The final coding frame will aim to reflect consensus among multiple members arrived at through our data clinics and discussion on key issues and findings.
